# A Smart and Multifaceted Mobile Health System for Delivering Evidence-Based Secondary Prevention of Stroke in Rural China: Design, Development, and Feasibility Study

**DOI:** 10.2196/13503

**Published:** 2019-07-19

**Authors:** Na Wu, Enying Gong, Bo Wang, Wanbing Gu, Nan Ding, Zhuoran Zhang, Mengyao Chen, Lijing L Yan, Brian Oldenburg, Li-Qun Xu

**Affiliations:** 1 Center of Excellence for mHealth and Smart Healthcare China Mobile Research Institute China Mobile Communications Corporation Beijing China; 2 Global Health Research Center Duke Kunshan University Kunshan China; 3 School of Population and Global Health The University of Melbourne Victoria Australia

**Keywords:** stroke, secondary prevention, rural health services, mobile application, software design, China

## Abstract

**Background:**

Mobile health (mHealth) technologies hold great promise in improving the delivery of high-quality health care services. Yet, there has been little research so far applying mHealth technologies in the context of delivering stroke care in resource-limited rural regions.

**Objective:**

This study aimed to introduce the design and development of an mHealth system targeting primary health care providers and to ascertain its feasibility in supporting the delivery of a System-Integrated techNology-Enabled Model of cAre (SINEMA) service for strengthening secondary prevention of stroke in rural China.

**Methods:**

The SINEMA mHealth system was designed by a multidisciplinary team comprising public health researchers, neurologists, and information and communication technology experts. The iterative co-design and development of the mHealth system involved the following 5 steps: (1) assessing the needs of relevant end users through in-depth interviews of stakeholders, (2) designing the functional modules and evidence-based care content, (3) designing and building the system and user interface, (4) improving and enhancing the system through a 3-month pilot test in 4 villages, and (5) finalizing the system and deploying it in field trial, and finally, evaluating its feasibility through a survey of the dominant user group.

**Results:**

From the in-depth interviews of 49 relevant stakeholders, we found that village doctors had limited capacity in caring for village-dwelling stroke patients in rural areas. Primary health care workers demonstrated real needs in receiving appropriate training and support from the mHealth system as well as great interests in using the mHealth technologies and tools. Using these findings, we designed a multifaceted mHealth system with 7 functional modules by following the iterative user-centered design and software development approach. The mHealth system, aimed at 3 different types of users (village doctors, town physicians, and county managers), was developed and utilized in a cluster-randomized controlled trial by 25 village doctors in a resource-limited county in rural China to manage 637 stroke patients between July 2017 and July 2018. In the end, a survey on the usability and functions of the mHealth system among village doctors (the dominant group of users, response rate=96%, 24/25) revealed that most of them were satisfied with the essential functions provided (71%) and were keen to continue using it (92%) after the study.

**Conclusions:**

The mHealth system was feasible for assisting primary health care providers in rural China in delivering the SINEMA service on the secondary prevention of stroke. Further research and initiatives in scaling up the SINEMA approach and this mHealth system to other resource-limited regions in China and beyond will likely enhance the quality and accessibility of essential secondary prevention among stroke patients.

**ClinicalTrial:**

ClinicalTrials.gov NCT03185858; https://clinicaltrials.gov/ct2/show/NCT03185858

**International Registered Report Identifier (IRRID):**

RR2-10.1016/j.ahj.2018.08.015

## Introduction

### Stroke in Rural China

China bears the largest stroke burden in the world. Every year, there are about 2.5 million new stroke cases, and 1 million deaths due to stroke [1]. According to recent estimates, the age-standardized prevalence of stroke in China was 1114.8 per 100,000 people, whereas the rate is significantly higher in rural areas where the prevalence has tripled in the past three decades [2]. Stroke prevention has become a national priority in China since 2009, and significant efforts have been made in establishing stroke registers, improving acute stage treatment, and screening high-risk populations [3]. However, accessibility and quality of secondary prevention of stroke is still a major public health challenge.

Currently, preventive care for stroke patients is suboptimal in rural China, and there is no clear strategy for improving secondary prevention of stroke in resource-limited settings in China and around the world [4]. The current lack of quality essential care provided by the health care system and the insufficient awareness on self-management among stroke survivors contribute to the high prevalence of stroke recurrence and stroke-related mortality in rural China. Village doctors and physicians in township hospitals, who undertake both the basic public health services and clinical services in rural settings, are primary care providers for delivering preventive care and health education for rural residents [5]. Most of them receive less than 5 years of professional medical training and are often unable to provide guideline-based high-quality health care services for stroke patients [6,7]. In addition, stroke patients generally lack awareness and education on secondary prevention, and previous studies found that adherence to medications for secondary prevention was poor among community-dwelling individuals after their discharge from the hospital [8]. To address these challenges, effective strategies for empowering the existing health workforce with the ability to deliver and promote secondary prevention of stroke are needed.

### Potential of Mobile Health

Owing to the rapid popularization of ubiquitous network connectivity and mobile phones, mobile health (mHealth) technology has emerged as a potential solution for improving both the accessibility and quality of health care, especially in resource-limited settings. There is an increasing body of evidence demonstrating that mHealth-based programs could improve health care service delivery and quality of care among people with chronic conditions [9-12]. However, there are still many challenging issues that remain to be addressed. First, most of these studies focused on health care providers who deliver clinical services at secondary or tertiary hospitals, so evidence is still very limited for mHealth programs designed for a primary health care context in resource-limited settings [10,13]. Second, although some recent research investigated factors related to adopting and using an mHealth system [14,15], most published programs appear to have relatively little input from the targeted end users [16,17]. Poor involvement of end users in the design stage likely leads to poor adoption of the new mHealth system and suboptimal usability and engagement in using the mHealth programs, which further limits the effectiveness of digital health interventions. Finally, the available mHealth programs usually provide care tools for one care role only rather than for a coordinated team of providers with multifaceted roles. Previous literature reviews revealed that except for individual factors, external factors, such as supporting the relationship with their coworkers and collaboration efforts at the organizational level, also facilitated the adoption of an mHealth system among health care providers, which is especially important for mHealth programs in developing countries [14,15].

### Study Objectives

To address these multiple determinants, we have designed an mHealth system for improving stroke care in rural China. This technology was used in the *System-Integrated techNology-Enabled Model of cAre* (the SINEMA project) for secondary prevention of stroke in rural China. The protocol of the SINEMA study has been published earlier [18]. In this paper, we will describe the design process of the SINEMA mHealth system as well as report the findings from the contextual research supporting the user-centered design, the iterative design and implementation of the SINEMA mHealth system, and the results from the users’ survey after a yearlong trial. The clinical effectiveness of the SINEMA intervention model is beyond the scope of this paper and will be discussed in a separate paper.

## Methods

### Study Site

The SINEMA study was undertaken in a region in China—Nanhe County, Hebei Province. This province, together with 8 other provincial regions, constitutes a high incidence *Stroke Belt* scattered from the north to west of China, which has a stroke incidence of 205.1 to 550 per 10,000 population, doubling that of the national average [19]. This study aimed to design, implement, and evaluate an mHealth model of care for secondary prevention of stroke. It was anticipated that the care approach would be generally applicable to other resource-limited settings in the country. Nanhe County is listed in the provincial government’s record as a *poverty-stricken county* with an annual disposable income per capita less than half of the national average of 11,030 RMB [20]. In Nanhe County, there are 2 county-level hospitals, 8 township hospitals (one for each township), and 218 village clinics (one for each village). Overall, 4 typical villages within the area were selected for contextual research and the pilot study.

### Design Framework of the System-Integrated Technology-Enabled Model of Care Mobile Health System

**Figure 1:**
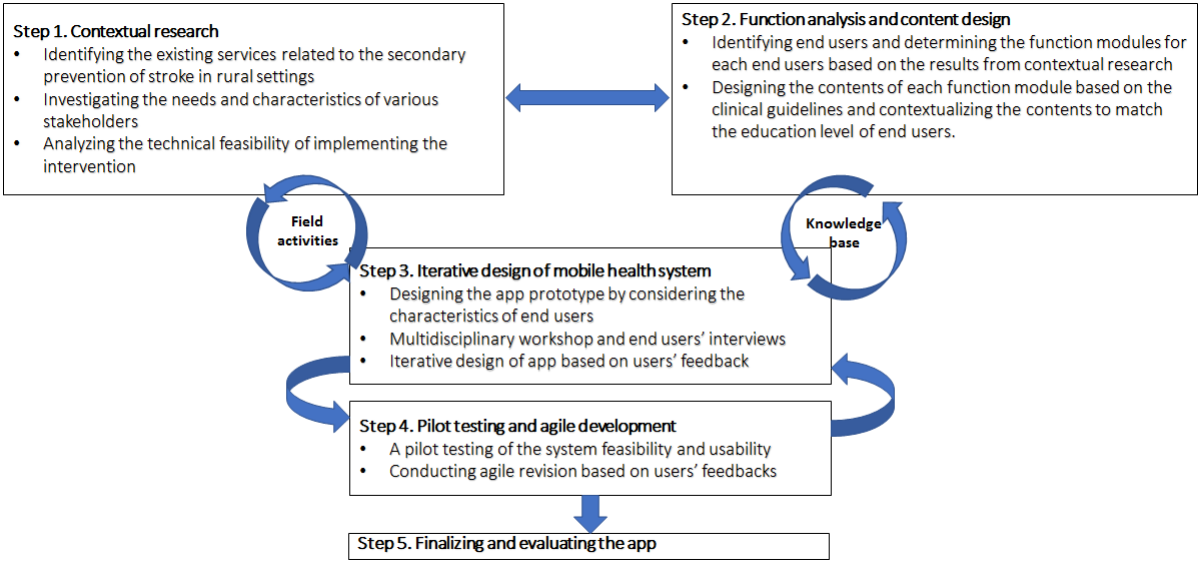
Steps involved in the design of the system-integrated technology-enabled model of care mobile health system.

In line with the user-centered design principles, our design framework consisted of 5 key steps, including (1) assessing the needs of relevant end users through in-depth interviews with stakeholders; (2) designing the functional modules and evidence-based contents based on end users’ needs; (3) iteratively designing and building the mHealth system structure and the app user interface based on end users’ roles and characteristics; (4) improving and enhancing the system through pilot testing and agile development based on end users’ feedback; and (5) finalizing the mHealth system, deploying it in field trial, and evaluating its feasibility through a survey of the dominant user group (summarized in Figure 1).

To successfully complete all these steps and tasks, we assembled a multidisciplinary team of researchers who have diverse expertise in public health, medical anthropology, behavioral science, stroke treatment, care transition, software design and development, and user interface design.

#### Step 1: Contextual Research and Needs Assessment

To design an effective system that caters to the needs of both health care providers and patients on secondary prevention of stroke, we first carried out contextual research in 4 villages in Nanhe County to identify existing health care services available and accessible in the villages and assessed the needs of relevant end users.

Considering the concerns from patients and families in discussing and sharing their experience with other people in the village, and participants’ geographically dispersed locations, we adopted face-to-face in-depth interviews as the most feasible method to collect data. Participants included stroke patients, family caregivers, village doctors, and health care providers in township hospitals. Participant recruitment occurred in collaboration with local health facilities, and stroke patients and their caregivers were referred by village doctors. The number of participants interviewed was decided based on a saturation point of obtaining a comprehensive understanding where no new substantive information was being acquired. The interviews were conducted by experienced researchers. Interviews followed preprepared semistructure interview guides, which were developed after discussions among the multidisciplinary research team (see Multimedia Appendix 1 for interview guides). Separate guides were used for different group of participants. Interviews were conducted in a private room (either in a private room in the clinics for health care providers or in the patients’ own house) to ensure the confidentiality of the information. All interviews were audio-recorded and transcribed verbatim in Mandarin Chinese.

#### Step 2: Functional Analysis and Content Design

**Figure 2:**
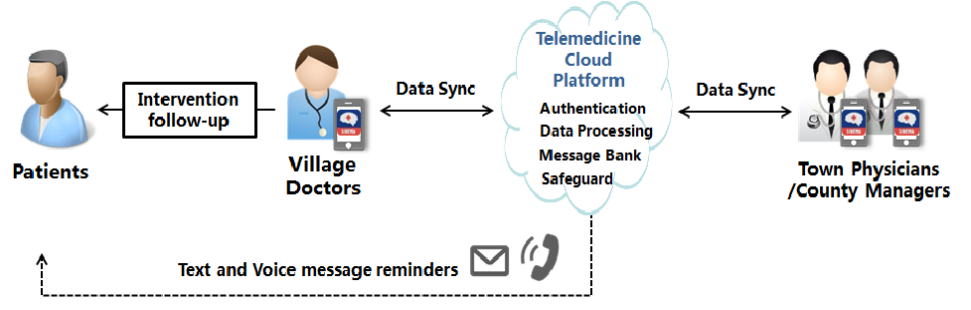
A schematic diagram of the system-integrated technology-enabled model of care mobile health system.

Based on of the results obtained from contextual research and the SINEMA intervention model design, the research team chose to design the mHealth system (Figure 2) in 2 parts, ie, an app and a cloud platform that is linked with a patient-oriented message dispatch system. The research team drafted a requirement document. In the document, types of end users were identified, including village doctors, township physicians, and county managers, with the latter two also assuming management roles. The overall aims of the mHealth system—empowering primary health care providers to perform guideline-based care for stroke patients and supporting them in delivering the SINEMA intervention method—were also clearly stated.

In this requirement document, the key functions and contents of the system modules were also drafted. The content of the modules was developed based on the Chinese clinical guideline for the secondary prevention of stroke in primary health care settings [21]. The guideline emphasizes on early diagnosis of stroke-related symptoms and risk factor management through lifestyle modification and a combination of medical therapies. On the basis of the guideline, stroke specialists proposed the structure of the follow-up visits, taking full consideration of the different types of strokes. Then a team of researchers translated the information from the guideline into a series of questions, following a standard logic flow, for village doctors’ follow-up visit. In line with the guideline, they also prepared training materials with videos, graphs, and texts, and a list of essential medicines to assist village doctors’ decision making on medication prescription. Behavior change techniques such as goals and planning, feedback and monitoring, and social support were applied to promote the actual behavior changes in end users. The contents were further simplified and contextualized based on the principles of meeting the basic education level of village doctors and availability of medicines in the villages. A paper-based demo version of the contents on follow-up visits, training materials, and medicine lists was then reviewed by stroke specialists. Using the clinical guideline as references, stroke specialists provided their feedback on contents during a workshop meeting and verified that the contents after the simplification process were in line with the clinical guideline recommendations after the simplification process.

#### Step 3: Iterative Design of the Mobile Health System

##### Design of the System Architecture

After clarifying the functions and modules, we designed the system architecture. The whole system comprised an app client and a cloud platform (Figure 2). The client was a 3-in-1 app for village doctors, township physicians, and county managers, which was running on an Android mobile platform, with different user interfaces and functional modules based on the type of roles assigned. The main reasons for choosing the Android phone were that a range of affordable phone models were available and that they were very popular among village doctors, which would facilitate future scale-up of the system in rural areas. The cloud platform contained 2 parts, the app server and database server. The app server had built-in functions of security authentication, services management, and data analytics as well as communication with the third-party voice and short messaging service (SMS) text messaging gateways. The database server was deployed in a protected network (private network) inaccessible to the public network, ensuring that the database service only opens the data ports to the app server.

**Figure 3:**
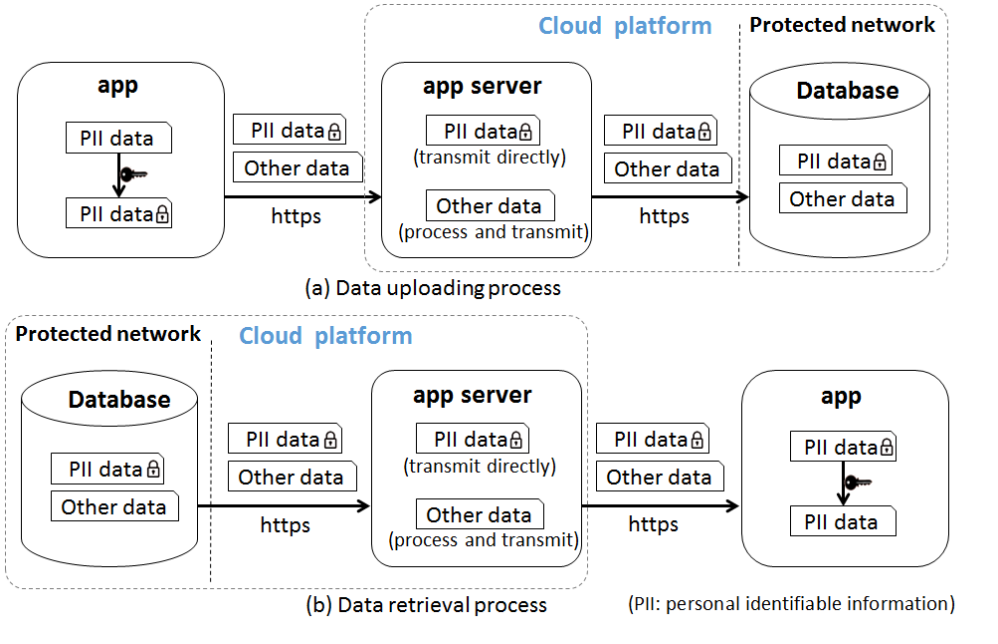
Information safety processing diagram.

Medical data and personal identifiable information (PII) are sensitive data concerning a user’s privacy that need to be transmitted over the network and stored in a secure manner to avoid data loss, breach, or malicious attacks [18]. PII data security, as one of the key issues raised by end users, was thoroughly discussed within the team and was emphasized in this system under the premise of architecture safety (Figure 3). In our system, to ensure the security of the data on the mobile phone, the app client did not store data [22]; all the data were obtained through the network, and they were automatically erased when the app was closed. For the data uploading process, first, the PII data were encrypted using Advanced Encryption Standard in the app with random keys, and then the https, a secure data transmission protocol, was used to transmit the data over the network; finally, the data were stored in the database server on the cloud. This dual method of encryption offered more effective data protection. Encrypted PII data were directly stored in databases, which enhanced the level of data storage security. Similarly, for the data retrieval process in which the app client requested for data stored in the database, the cloud transmitted the encrypted PII and other data requested to the app client to be used by the end user. As the app server needed to obtain the plain text of a patient’s phone number for sending an SMS text messaging, the phone number was not encrypted on the app client but encrypted on the app server.

We designed the SINEMA mHealth system with the methods of modularization [23] and good encapsulation to implement separate service modules and commonly used components [24] of tools to meet the iterative design and development needs [25].

##### User Interface and User Experience Design

The user interface should be designed to be friendly and efficient, which is the key to improving users’ experience in utilizing and engaging with the app. Our team has designed a human-centered app by following the principle of a friendly and efficient user interface with thorough consideration of users’ needs and characteristics [26].

First, the user interface and user experience design should reflect users’ mental models. As our target users are professionals, including doctors from village clinics and from township and county-level hospitals as well, the design of the interface took full account of their respective needs and expectations as well as limitations. In line with the results from the contextual research, we assessed the users’ proficiency in using smartphones by choosing an extensively used app as a benchmark. Considering that county and town hospital doctors are already familiar with Web-based health care information system, we adopted similar structure design and visual styles.

Second, the design should improve workflow efficiency, avoid information-entry errors, and provide smooth app access for users. Therefore, we designed simple and easily recognizable icons, such as contrasting colors for positive and negative information and distinctive graphical shapes to indicate different click status, to quickly draw users’ attention and help them understand the information effectively. In addition, we used uniform marks for navigation to make the transition and interaction of different interfaces clear and consistent. Designing with specific blocks to distinguish information contents and prefilling with defaults also improved the usability and reduced the chance of entry errors.

#### Step 4: Pilot Testing and Agile Development

Following the above studies, we then developed a fully functional app prototype, which was used for discussion in the multidisciplinary workshop as well as for demonstration to and interviews with the village doctors. On the basis of their feedback, we carried out the first round of iterative design and developed a testing version for the pilot study.

The pilot study was conducted in 4 rural villages of Nanhe County for 3 months. During the pilot study, each village doctor was provided with an Android phone with the app installed to implement the SINEMA intervention model. Each village doctor was asked to care for about 10 patients in his or her village. In-depth interviews with all the village doctors and township physicians were conducted after the pilot study to collect any feedback about the usability of the app and suggestions to optimize the app.

On the basis of the feedback from the end users, we conducted several rounds of revision of the app. During the process of development, we took advantage of the latest technologies to speed up the iteration, for example, the online tracking and positioning of bugs for rapid and efficient feedback and the hotfix technology for repairing and updating the app without releasing a new version of the app for village doctors.

#### Step 5: Main Trial and Users’ Follow-Up Survey

The finalized app system was utilized to support the implementation of the SINEMA method in 25 villages, which had been randomized into the intervention arm of a cluster-randomized controlled trial covering 50 villages in 5 townships. The published protocol paper contains details regarding the trial design [18]. Village doctors in the intervention arm were invited to complete a survey through qualtrics.^xm^ (Qualtrics) online platform after the 1-year intervention. The survey included questions related to village doctors’ demographic characteristics and their experience of implementing each component of the SINEMA method including this mHealth system. Village doctors were asked to report their agreement level with the statements regarding the usability and satisfaction on various functions of the SINEMA app according to the 5-level Likert scale, ranging from very disagree to very agree.

### Analytical Approach

Verbatim transcriptions in Chinese were analyzed using a thematic analysis [27]. Researchers first read through all transcripts to be familiar with the data. Themes were then developed based on the interview guide and discussion among researchers to capture the important features of the data. Codes were generated for each transcript line by line and grouped into categories. Each category was then reviewed and examined again, and groups of main categories were refined before finalizing the major themes. Coding process and key themes were discussed within the research team. Coding was performed by WG and reviewed by EG, followed by a discussion to ensure the trustworthiness. Quotations used in this paper were translated from Chinese to English and then back-translated into Chinese to increase the transparency of the interpretation. NVivo 11 qualitative analysis software (QSR International) was used to facilitate the coding approach.

The survey data in this study were analyzed by using STATA statistical software (Version 15, StataCorp LLC). A descriptive analysis was performed, and frequencies and proportions were reported for categorical variables.

### Ethical Considerations

The contextual research and pilot study were approved by the Duke Kunshan University Ethical Committee. The main SINEMA trial was approved by the institutional review boards of the Duke University Health System in the United States and Tiantan Hospital in Beijing, China. The trial was registered in ClinicalTrials.gov (#NCT03185858). All participants provided their written informed consent before the study began.

## Results

### Findings From the Contextual Research

In total, our team visited the study sites 3 times during the system design stage and conducted 49 in-depth interviews with various stakeholders, including 5 physicians at township and county hospitals, 12 village doctors, 22 stroke patients, and 10 family caregivers. On the basis of the analyses of the qualitative data from 17 in-depth interviews (12 village doctors and 5 township hospital physicians), we generated 4 broad themes related to the development of function modules. The data from 22 patients and 10 family caregivers were considered as a benchmark to identify the gaps between the existing and expected care services and were mainly used for informing the development of a messages-dispatching system, which will be reported in another paper.

#### Theme 1: Lack of Awareness and Knowledge on the Secondary Prevention of Stroke Among Village Doctors

On the basis of the interviews, most village doctors could identify stroke patients in the villages, but they were not able to provide effective preventive services for this special patient group. Most village doctors were not aware of the guideline on the secondary prevention of stroke and received none-to-minimum training related to stroke prevention. The insufficient awareness and knowledge on guideline-recommended pharmacological treatments and physical rehabilitation limited the services that village doctors could provide when stroke survivors failed to adhere to the essential medications prescribed at discharge or failed to do physical exercise and rehabilitations. The services that village doctors provide to stroke patients are mainly covered by the basic public health services, which focus on the management of hypertension and diabetes as risk factors.

#### Theme 2: No Existing Electronic Record System for Managing Patients’ Visits in the Villages

During the interview, several village doctors mentioned that they were not able to keep records of the patients’ visits because there is no electronic record system; most of their work is still paper based. The existing electronic systems, including a system for purchasing medicines and a system for uploading records of basic public health services, were all developed by the government and are recently being utilized by village doctors. However, these electronic systems were not equivalent to the hospital information system; thus, they were unable to support village doctors to manage patients’ routine visits. A few village doctors mentioned that they took paper-based notes to record patients’ visits:

We do not have electronic record system to record patients’ visit in the clinic...I sometimes write the prescription on paper sheets, but actually I seldom review these paper sheets because the note is not kept systematically.

Village doctor

Owing to the lack of an electronic record system, the decisions made by village doctors were mainly based on unreliable recalls from patients and their family members:

These patients or their family members come to my clinic and tell me what medicines they want to purchase, and I will prescribe based on that.

Village doctor

#### Theme 3: Township Physicians’ Supports and Supervisions Among Village Doctors

During the interview, several village doctors mentioned that their performance was supervised by township physicians who visited village clinics and checked paper-based records for the basic public health services as indicators for village doctors’ performance. Township physicians also mentioned their roles in supporting and managing village doctors:

We are adopting the integration of management between township and villages. We have very close communications with village doctors. We organize monthly meetings among village doctors to provide updates mainly on basic public health services. We also invite village doctors to take part in trainings organized by county hospitals or the county health bureau.

Township physician

#### Theme 4: Great Interest in Applying Mobile Health Technology in Daily Work but the System Should Be Simple to Learn and Use

Village doctors were also interviewed on their use of smartphones and the availability of internet in the clinic. All village doctors we interviewed owned a smartphone and were accustomed to using multimedia apps. Most of the village doctors we interviewed mentioned that their clinics have good internet access and some of the clinics have Wi-Fi. However, only a few village doctors had actually installed health-related mobile apps to support their daily work, and the key concern was the simplicity and ease of use of the apps:

I am interested in using the app for managing stoke patients if it could make things more convenient, but if the APP is too complicated, then I won’t use it.

Village doctor

### Functional Modules of the System-Integrated Technology-Enabled Model of Care Mobile Health System

**Figure 4:**
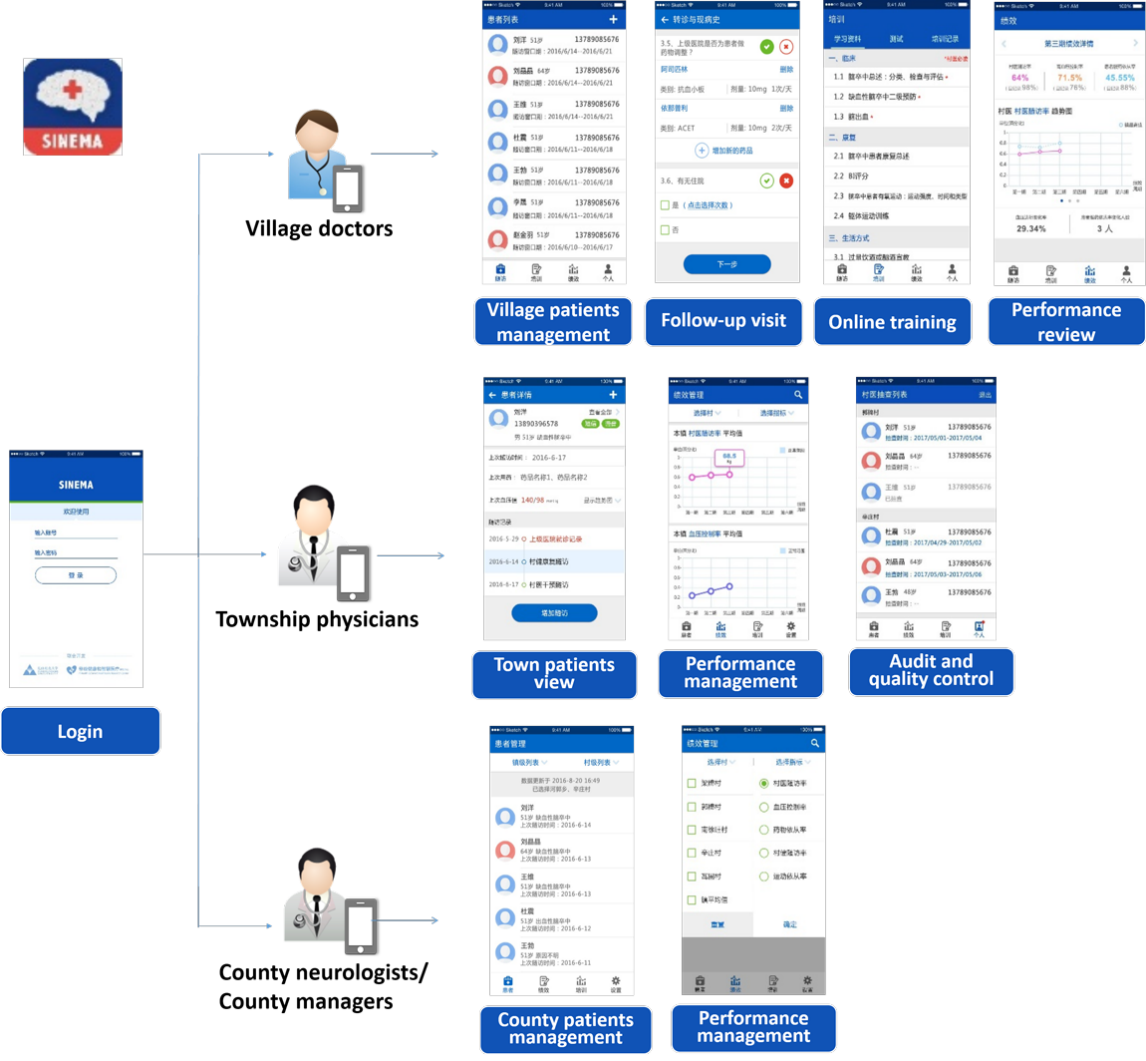
The screenshots showing the functional modules of system-integrated technology-enabled model of care mobile health app for the 3 types of end users.

The findings from the contextual research and the targeted activities required by the SINEMA intervention model were translated into a needs document, which suggested the potential functional modules and the contents within each module. A multidisciplinary workshop was organized, and the functional modules were refined based on the end user’s feedback. Details for each functional module are described below and shown in Figure 4.

- *Patient Management:* This module enabled village doctors to add basic information of stroke patients in the system and helped them reach the patients easily through phone calls. Village doctors and township physicians were also able to search for the records of patients within their responsible geographical areas.
- *Follow-up visits:* This module was designed for village doctors to record their patients’ follow-up visits. The contents were translated and contextualized based on the clinical guidelines and were verified by stroke specialists, which enabled village doctors to communicate with patients based on a standard workflow consisting of evaluating symptoms, measuring blood pressure, asking about medication compliance, recording newly prescribed medicines, and uploading other supportive paper-based materials. In addition, the app would pop up alerts for special attention and referral if the recorded patients’ status was abnormal.
- *Online training:* To enhance the knowledge and skills of village doctors, we designed a module for online training. The training materials were in line with the clinical guidelines but simplified by stroke specialists based on the knowledge level of village doctors. The training materials covering the topics of stroke treatment, rehabilitation, and lifestyle modification were displayed suitably in the format of videos, graphics, and texts. The training modules also contained quizzes to help village doctors evaluate their self-learning progress.
- *Reminders:* To facilitate the work planning of village doctors for their patients’ monthly follow-up visits, we designed the function of reminders with 3 types of presentations: an independent follow-up reminder calendar, the next follow-up reminders on the patients list, and the daily pop-up reminders of a list of follow-ups required for a particular day.
- *Performance review and management:* To monitor the progress of each stroke patient and to motivate village doctors to achieve their performance indicators of the intervention effectively, we designed a performance management module. This module displayed in real time the summary performance data including the follow-up rate, the blood pressure control rate, and the medication adherence rate among all stroke patients under a village doctor’s management. The township and county mangers could also review these indicators and make comparisons across all villages and townships of their respective responsibilities.
- *Random audit and quality control for township physicians:* To facilitate the supervision role of township physicians, we designed the random audit module for township physicians. Each month, the system will randomly select 3 patients for township physicians to conduct auditing phone calls.
- *Integrating with SMS text messaging and voice messaging system:* To fulfill the intervention need of delivering health education information via messages to stroke patients, the patients’ list was linked with the message dispatching system.

### Iterative Modular and User Interface Design

**Figure 5:**
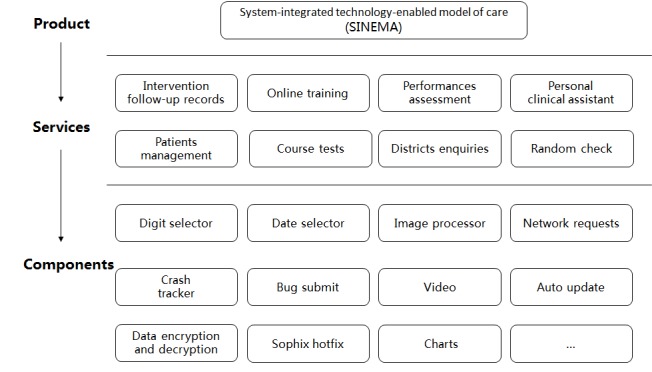
Modular design of the Android app architecture.

We translated the functional modules into a practical mHealth app by following the modular design principles of high cohesion, low coupling, and good scalability (see Figure 5) [28,29]. We addressed several technical difficulties throughout the system design and development process. First, to fulfill the needs of multirole end users, including village doctors, township physicians, and county managers, we implemented a complex service logic, which enabled the SINEMA mHealth app to display different functions and data on the same page according to the role of the users. Second, we designed a data structure of stack to store all the data for every node at all layers to ensure the flexibility of the intervention follow-up for village doctors. By doing so, we were able to avoid the long workflow and prevent function error on page jumps, cross-page data transfer, and page fallback. Finally, to achieve the media features, including taking pictures, image processing, and online video download and playing, we compressed images effectively with a compression rate of 94%, which could reduce system memory overhead, power consumption, and the consumption of mobile data traffic. In addition, iterative development could be accelerated through adopting the latest open-source technologies. Depending on the resources available in the deployment environment, the mHealth system server is able to support secure access from 100,000 users.

On the basis of the results from analyzing the most popular apps among village doctors, we adopted the style of social interactions and linear path for the user interface design. As displayed in Figure 4, we designed simple and intuitive icons and applied consistent gestural design (taps or swipes or pinches or scrolls) to increase the functionality of the app. We also displayed information in various formats, including plain texts, choice questions, graphs, and videos, to strengthen the engagement of the app. In addition, the design was further refined based on feedback from end users. For example, we simplified the information briefing on the patients’ list page and added a ranking mode to the patients’ list to help end users find the right record more efficiently.

### Pilot Trial and Revision Based on Iterative Design

Before using the mHealth system on a large scale, we tested the system and the intervention model through a pilot trial. A total of 4 village doctors in Nanhe County, from 4 villages, were equipped with the SINEMA mHealth app to manage a total of 54 patients for 3 months. After the trial, we interviewed the end users to collect their feedback about the SINEMA model and the mHealth system design. We had since refined the mHealth system and generated 4 iterative versions of the system to better cater to the local situation. Examples of improvements included adding a detailed list of medication guidelines in the follow-up module to support village doctors’ decision making during follow-up visits, adjusting the algorithm of message sending by considering the low literacy rate among stroke patients, refining the contents of health education by emphasizing on physical activity and medication adherence, revising the calculation methods of performance indicators, and adding the module of phone-call auditing for township physicians to fulfill the needs of quality control of the intervention. The final version of the SINEMA mHealth system was granted a software copyright register from the China National Copyright Administration (Registration Number 2017SR502445).

### Survey Results on the Feasibility of Using System-Integrated Technology-Enabled Model of Care Mobile Health System

During July 2017 and July 2018, the SINEMA mHealth system supported 25 village doctors, 5 township physicians, and 2 county managers in managing 637 stroke patients. After the 1-year trial, 24 out of 25 village doctors (response rate=96%, including 4 females) completed the survey. On average, they were 46.5 years old (SD 7.9) and had 24.4 years (SD 8.8) of clinical service. Table 1 shows the results from the survey. All participants self-reported that the app was easy to learn and use, and 22 out of 24 (92%) reported that they did not have any technical or functional issue during the yearlong usage. Over two-thirds of respondents strongly agreed that the app was able to standardize their workflow of the follow-up visits (71%) and help them make the right decision (67%). Most of them regarded that the functions and tools enabled them to easily connect with their patients, review patients’ history, and share information with township physicians, which were very helpful features. Overall, 22 village doctors (92%) strongly agreed or agreed that they would like to continue using the app after the trial.

**Table 1:** Village doctor’s survey responses at 12 months of the main trial (N=24).

Village doctors’ demographic characteristics and feedback			n (%)
**Demographic characteristics**			
	**Gender**		
		Male	20 (83)
		Female	4 (17)
	**Age (years)**		
		30-39	4 (17)
		40-49	11 (46)
		50-59	7 (29)
		60-69	2 (8)
	**Education level**		
		High school or equivalent	13 (54)
		Community college	8 (33)
		College degree	3 (13)
	**Years of being a village doctor**		
		1-10	3 (13)
		11-20	4 (17)
		21-30	12 (50)
		30+	5 (21)
**Agreement toward statements related to System-Integrated Technology-Enabled Model of Care app**			
	**I spent very short time in learning how to use the System-Integrated techNology-Enabled Model of cAre app**		
		Strongly agree	18 (75)
		Agree	6 (25)
	**I did not** **have** **any technical or functional issue during the 1-year usage**		
		Strongly agree	11 (46)
		Agree	11 (46)
		Neither agree nor disagree	2 (8)
	**This app helped me connect to my patients easily**		
		Strongly agree	15 (63)
		Agree	8 (33)
		Disagree	1 (4)
	**This app helped me standardize patients' follow-up visit**		
		Strongly agree	17 (71)
		Agree	6 (25)
		Neither agree nor disagree	1 (4)
	**I reviewed the patients’ previous records in the app during the follow-up visit**		
		Strongly agree	18 (75)
		Agree	5 (21)
		Neither agree nor disagree	1 (4)
	**This app support decision making for medication prescription**		
		Strongly agree	16 (67)
		Agree	8 (33)
	**Sharing information with township physicians through this app is important for me**		
		Strongly agree	10 (42)
		Agree	12 (50)
		Neither agree or disagree	2 (8)
	**I am willing to continue using this app after the trial**		
		Strongly agree	10 (42)
		Agree	12 (50)
		Neither agree or disagree	2 (8)

## Discussion

### Principal Findings

This paper described in detail the design and development of the SINEMA mHealth system and reported the results from the users’ survey. To research and develop this mHealth system, our multidisciplinary research team applied various research methodologies, adopted an iterative design approach, and involved end users throughout the whole process. The SINEMA mHealth system featuring 7 distinctive functional modules has fulfilled its intended goal in supporting the delivery of the SINEMA intervention method. Building upon the mHealth technology, the system has demonstrated the potential to empower village doctors to carry out patient management, self-training, and follow-up visits. The system is also able to facilitate township and county managers in auditing and supporting village doctors. The survey among the dominant group of end users (village doctors) revealed that most of them were satisfied with the functions of the mHealth system in formalizing the follow-up visits and improving clinical skills, and they were keen to continue using the system after the trial.

The overall structure and individual functional modules of the SINEMA mHealth system were designed to align with the distinct roles of end users in the conventional health care system. A review of mHealth intervention studies in China indicated that most previous studies focused on patient education and behavior change, with almost no work centering on interprovider communications and health services management [30]. Previous studies also revealed that the key enabling factors for an mHealth system’s adoption and use [14] are organizational support environment, facilitating collaboration between coworkers, and integration with health care system. With a view to integrating the existing health care protocol with more active interactions across different tiers of health care providers, we designed a single system catering to 3 types of health care providers: village doctors, township physicians, and county managers. To fulfill their respective needs, various functional modules were designed based on the scope of their work. On the one hand, shared functional modules, such as the patients’ management module and performance management module, promoted an active and efficient information exchange among end users. On the other hand, the functional modules on training and follow-up visits were specifically for village doctors to learn relevant medical knowledge and skills and to perform follow-up services. Therefore, the introduction of the SINEMA mHealth system with both shared information and specific functional modules among 3 types of end users supported the delivery of the SINEMA intervention model that promotes the integration of the health care system across the village, township, and county levels.

In addition to the structure of the mHealth system, the contents of each functional module have been designed by fully considering the needs of the end users. Although studies on using digital health technologies for preventing and controlling chronic diseases have proliferated in recent years, the evidence of their effectiveness is not abundant. Studies have found that the levels of user engagement greatly impact the outcomes of the interventions [31,32]. These findings support a user-centered design approach to developing digital health solutions [33]. In our study, we involved end users throughout the whole development process. We assessed the end users’ needs and work patterns through in-depth interviews and simplified the contents of training and follow-up visit modules to match their knowledge levels. In addition, we took account of the end user’s phone use habits in user interface design and iteratively refined the system based on their feedback and comments. The highly intensive involvement of end users in both content and interface design ensured the high usability of end users in the SINEMA study and led to high satisfaction among users, as reflected in the survey results.

It should be noted that this mHealth system resulted from the concerted efforts of an international multidisciplinary team including public health researchers (medical anthropologists, public health practitioners, and behavior scientists), clinical practitioners (stroke specialists, rehabilitation therapists, and clinical pharmacists), and information technology experts (product manager, user interface designers, app platform architects, and software engineers). Collaborations among experts of different disciplines are never easy [34,35]. Inherent differences in domain terminology, priorities, and work cultures may create barriers for effective collaborations. Sharing of common goals and a mutual understanding are essential but not adequate to overcome these barriers. Frequent communications, patience, and persistence are also needed to ensure the success of the collaboration.

### Strengths and Limitations

This mHealth system is innovative in several ways **.** First, to the best of our knowledge, it is the first one ever built to deliver quality essential care on secondary prevention of stroke in rural China. The development of the SINEMA mHealth system provides a novel technical solution to address the increasing burden of stroke in China. The SINEMA mHealth system attempts to strengthen the primary health care system in terms of health care service delivery, health workforce, and health information system as the building blocks of the health system [36]. By equipping grassroots health care providers in the rural communities with the SINEMA mHealth system, the intervention has the potential to increase the delivery of and access to high-quality services related to secondary prevention of stroke, to improve the communication and task sharing among providers from tiered institutions, and to establish a health records system for stroke patients’ management and follow-up. Second, the SINEMA system is one of the very few mHealth systems that enable patient engagement and behavior changes at individual levels, as well as novel health care delivery models in the era of a connected world. Although the digital health industry expanded fast in China in the past years, the development of a digital solution for public health interventions remains premature, with little exploration of how a digital solution could be adopted by the existing health care system [30]. Finally, multiple technology design methods have been adopted, such as user-centered design, IT system research framework, supported by a multidisciplinary research team. This study adds new practical knowledge to the field in developing a digital health solution to address real-world public health challenges.

Our study and the mHealth system also have some limitations. We were able to involve end users’ feedback and conduct a pilot testing but were not able to conduct a comprehensive evaluation on all aspects of the mHealth system. However, according to the survey results among all the village doctors, they highly valued the usability of this mHealth system with a strong willingness to continue using the app after the trial. The system can be further improved along several directions. For example, artificial intelligence-based voice technology can be used to operate a more complex algorithm that enables dispatching personalized voice messages based on the updated medical records by village doctors. In addition, better integration of the mHealth system with the existing public health system and local hospital electronic medical record system is desirable. Such integration can facilitate the information exchange across health care facilities, reduce the duplicated workloads of village doctors, and promote a patient-centered continuum of stroke care.

### Conclusions

In summary, the SINEMA mHealth system, designed in a user-centered and iterative way by a multidisciplinary team, achieved high functionality, feasibility, and user satisfaction among village doctors in rural China. The evaluation of the clinical effectiveness of the system to improve patient outcomes is currently being undertaken. If proven effective, the adoption and scaling up of the system to more areas for a longer term has the potential to reap large public health benefits through improving quality of primary care and reducing disease risks from stroke. The system can also be adapted to managing other chronic conditions such as hypertension, diabetes, or heart disease. In future iterations, newer technology features and better integration with the existing digital health system will further enhance its impact.

## Appendix

Multimedia Appendix 1 Semistructured interview guides.

## Abbreviations

mHealth: mobile health
PII: personal identifiable information
SINEMA: System-Integrated techNology-Enabled Model of cAre
SMS: short messaging service
