# Supplementary material for: A Smart and Multifaceted Mobile Health System for Delivering Evidence-Based Secondary Prevention of Stroke in Rural China: Design, Development, and Feasibility Study
Source: JMIR Mhealth Uhealth. 2019 Jul 19;7(7):e13503. doi: 10.2196/13503 (PMC6676792; doi:10.2196/13503)
Supplement: Multimedia Appendix 1 [file mhealth_v7i7e13503_app1.pdf]

Appendix 1: semi-structured interview guides

Interview guide and question examples (Village Doctors)

Topics	Examples of questions
**Doctor information**	
○ Age and gender	• What is your age?<br>• What is your zodiac animal?
○ Residence	• Are you from local? Born and/or raised here?<br>• Do you currently live in this village?<br>• How many days of a year do you spend outside the village?
○ Education	• What is your highest degree?<br>• Where were you trained to become a village doctor?<br>• How often do you receive training from the township and county-level facilities?
○ Daily work	• How long have you been working as a village doctor?<br>• Are you the only doctor in this village? How many more?<br>• How many hours do you work every day?<br>• How many patients do you see every day? Among them, how many are for diagnosis, or for simple check-ups (e.g. blood pressure)?
○ Employment	• Are you appointed by the village, town, or county?<br>• To whom do you directly report?<br>• What amount to your monthly income?<br>• Is your salary affected by your performance as a village doctor?<br>• Who evaluates your performance? On what aspects?
**Village information**	
○ Demography	• How many people and households are in this village?<br>• What is the age structure like?
○ Transportation	• How long does it take to travel to the town, and the county?<br>• Is there any public transportation available?
**Stroke**	
○ Stroke prevalence	• How many people have a history of stroke in the village?<br>• How many new cases of stroke happened in the last 12 months?<br>• How many reoccurring cases of stroke happened in the last 12 months?
○ Medication	• Do stroke patients usually get their medication from the clinic?<br>• How do you prescribe the medicine for the patients?<br>• Can patients take their medication as directed (amount and duration)?<br>• If you have questions regarding medication, how would you seek for opinions or instructions from other medical professionals?
○ Medication cost	• How much do stroke patients usually pay for their medication monthly?<br>• Are there any patients who stop or reduce medication because of the unaffordability of the cost?<br>• How much of their medication expenses can be subsidized by the government-related insurance/support?

○ Awareness	• Do you know stroke secondary prevention?<br>• Do you know the guideline?
○ Rehabilitation	• Do you usually give the patients instructions for rehabilitation?<br>• Do patients ask you about rehabilitation?<br>• If you are equipped with the knowledge and skills to help the stroke patients to rehabilitate, would you have time and interests to do it?<br>• Can you suggest anyone who can be trained to be a village health promoter to help patients rehabilitate?
○ Medical check-up	• How often do stroke patients come to the clinic to check their blood pressure?<br>• Do patients bring their hospital test results to you when they return to the village? Do you adjust your prescription based on the test results?
○ Management	• Do you have any system to manage the stroke patients?<br>• What are the problems you have to manage these stroke patients?
○ Interest in APP	• Do you use and medical APP?<br>• Are you willing to use an APP to manage patients?<br>• Any suggestions on the design of the APP?
**Motivation**	
○ Eagerness to know	• How often do you ask patients (of any sorts) to come the clinic for check-ups, or to know about their progress?<br>• How often do you recommend them for a physical exam at a hospital?<br>How often do you ask the patients regarding their medication adherence?
○ Eagerness to learn	• How often do you attend professional trainings to improve your clinical capacity?<br>• How often do you seek out medical knowledge from other sources?<br>How often do you consult other doctors, especially the ones from the township and county-level hospitals on medical cases?
○ Intrinsic motivator	• Do you think stroke patients' health status would improve if they closely follow the right medical instructions and advices?<br>• Do you know any examples in the village?
○ Extrinsic motivator	• Is your pay/income affected by your patients' progress (treatment and recovery)?<br>• Would you care more if there were more incentives?
**Others**	
○ Cellphone use	• Do you own a cellphone?<br>• Do you use it often? Phone calls, text-messaging?<br>• Do you know how to use Wechat?
○ communication	• Do you communicate with township managers or other villager doctors regularly?<br>• What do you usually communicate?<br>• Do you have any problems to communicate with them?

Interview guide and question examples (Township Physicians)

Topics	Examples of questions
**Doctor information**	
○ Age and gender	• What is your age?<br>• What is your zodiac animal?
○ Residence	• Are you from local? Born and/or raised here?
○ Education	• What is your highest degree?<br>• Where were you trained to become a township doctor?<br>• How often do you receive training higher-level facilities?
**Stroke**	
○ Stroke prevalence	• How many people have a history of stroke in the township?<br>• How many new cases of stroke happened in the last 12 months?<br>• How many reoccurring cases of stroke happened in the last 12 months?
○ Management	• Do you or village doctors have any system to manage the stroke patients?<br>• What are the problems you have to manage these stroke patients?
○ Interest in APP	• Do you or village doctors use and medical APP?<br>• Will village doctors be willing to use an APP to manage patients? And challenges?<br>• Any suggestions on the design of the APP?
○ Trainings	• Do you provide any trainings to village doctors on stroke?<br>• Do they have any others trainings on stroke?
**Compassion**	• How much do you care about the improving the health status of stroke patients?<br>• How much do you think something should be done to improve the health status of stroke patients?
**Motivation**	• Do you think stroke is one of the major health concerns of the village?<br>• Do you think the health status of stroke patients would improve if they can strictly follow the instructions of an efficient medical intervention practice?<br>• Do you have any examples to share?<br>• Would you be motivated to contribute to the SINEMA project?
**Suggestion**	• What suggestions/advices can you offer to the SINEMA project?<br>• Which part of the project do you like the most?<br>• What do you think is the biggest obstacle of its success?

Interview Guide and Question Examples (Stroke Patients)

Topics	Examples of Interview Questions
**Patient information**	
• Age and gender	○ What is your age?<br>○ What is your zodiac animal?
• Marital status	○ Are you or have you been married?
• Current occupation	○ What do you do for a living now?<br>○ What were your previous jobs?
**Household information**	
• Cohabitants	○ Who live with you?
• Caregiver	○ Who takes care of you on a daily basis?
• Income	○ What is the primary source of income?
**Overall health status**	
• Diagnosed illnesses	○ Have you ever been diagnosed with hypertension, heat attack or other chronic conditions?<br>○ Have you had stroke before?
• Mobility	○ Can you stand up or walk?<br>○ Do you need a walking stick or assistance from others?
• Vision and hearing	○ Do you see and hear clearly?
**Stroke**	
• Stroke history	○ When was the first time you had stroke?<br>○ How old were you when you had the first stroke?
• Stroke reoccurrence	○ Has it recurred since the first time?<br>○ How many times has it recurred? When?
• Stroke subtypes	○ Was it an ischemic or a hemorrhagic stroke?
• Hospitalization	○ Were you hospitalized during the stroke?<br>○ Which hospital were you treated at?<br>○ How much did it cost when you were hospitalized for stroke?
• Previous health conditions	○ Had you been diagnosed with hypertension or any other cardiovascular diseases before you had stroke?<br>○ Were you a physically fit person before the stroke?
**Post-stroke repercussions**	
• Cognition	○ Was your cognitive ability affected by stroke?
• Speech	○ Was your ability to speak affected by stroke?
• Mobility	○ Was your ability to move affected by stroke?
• Vision and hearing	○ Was your ability to see and hear affected by stroke?
• Digestion	○ Was your appetite and digestive function affected by stroke?
• Incontinence	○ Did you start experiencing urinary and/or bowel incontinence after the stroke?
• Recovery	○ How much have you recovered from these repercussions?

**Medication**	
• Types of medication	○ How many types of medication do you take on a daily basis?<br>○ How many are for stroke treatment?
• Awareness	○ Do you know the purpose of your medication?<br>○ Do you realize the importance of your medication?
• Prescriber	○ Who prescribed them?
• Provider	○ Where do you purchase them?
• Medication adherence	○ Do you take your medication as prescribed?<br>○ How often do you forget to take your medication?
• Professional advice	○ Have you been told by doctors about the importance of medication adherence?
**Activity**	
• Physical activities	○ How much time do you spend on tending crops?<br>○ How much time do you spend on walking or biking?<br>○ Did stroke affect your activity level?
• Sedentary activities	○ How much time do you spend on sitting or lying?<br>○ Did stroke increase your sedentary time?
• Self-reliance	○ Can you and do you cook?<br>○ Do you need help when using the bathroom?
• Professional advice	○ Have you been told by doctors about the importance of exercising?
**Diet**	
• Digestive conditions	○ Do you have any digestive problems?
• Vegetable consumption	○ How often and how much do you eat vegetables?<br>○ Did you start eating more vegetable after the stroke?
• Fruit consumption	○ How often and how much do you eat fruits?<br>○ Did you start eating more fruit after the stroke?
• Sodium intake	○ Do you think your diet is salty, or saltier than others'?<br>○ Did you start eating less salt after the stroke?
• Professional advice	○ Have doctors told you about the importance of healthy eating?
**Tobacco and alcohol**	
• Smoking	○ Do you smoke on a daily basis? Did you smoke before?<br>○ Did stroke affect your smoking habit?
• Drinking	○ Do you drink on a daily basis? Did you drink before?<br>○ Did stroke affect your drinking habit?
• Processional advice	○ Have doctors told you by doctors about the importance of quit smoking and reducing alcohol consumption?
**Needs and expectations**	
• Medication	○ Do you think your current medication is effective?<br>○ Are you experiencing any side effects?<br>○ Do you think any of your medication is unnecessary?<br>○ Do you have trouble taking medication as prescribed?
• Activity	○ Do you know how to maintain a healthy level of activity?<br>○ Do you have trouble exercising at a desired or a required level?

• Diet	○ Do you know what a healthy diet is?<br>○ Do you have trouble following a healthy diet?
• Smoking and drinking	○ Do you have trouble cutting back smoking or drinking?<br>○ Do you know by how much to reduce drinking?
• Financial burden	○ Were you/your family able to pay the medical when you were hospitalized for stroke?<br>○ Can you afford the medication you have now?<br>○ Were your family income affected by your stroke?
• Mental burden	○ Are you mentally stressed because of your illness?<br>○ Do you think your personality has changed after the stroke?
• Expectations	○ Among all the repercussions caused by stroke, which do you wish to recover from the most?<br>○ Based on your recovery progress so far, what improvement do you expect in the next 12 months?
**Motivation**	
• Health status awareness	○ How often do you check your blood pressure, blood sugar, and blood lipids?<br>○ How often do you get physical exams?<br>○ How often do you consult with a doctor on your health status?
• Eagerness to learn	○ Do you often look for health-related knowledge (e.g. via news, books, podcasts, etc.)?<br>○ Do you often discuss your illness and other health related issues with your relatives and neighbors?
• Patient compliance	○ Do you always follow doctors' medical instructions and advices?<br>○ How often do you make your own decisions on medication without consulting a doctor?
• Intrinsic motivator	○ Do you think your health status would improve if you closely follow the right medical instructions and advices?<br>○ Do you know anyone who had similar heath status/illness as yours but has improved since?
• Extrinsic motivator	○ Do your family think your health status would improve if you follow the right medical instructions and advices?<br>○ How much do your family depend on you in terms of providing labor and income?
**Others**	
• Literacy level	○ Can you read and/or write?
• Cellphone use	○ Do you own a cellphone?<br>○ Do you use it often? Phone calls, text-messaging?<br>○ Do your caregiver, or other family members use cellphones?
